# Anti-Biofilm and Associated Anti-Virulence Activities of Selected Phytochemical Compounds against *Klebsiella pneumoniae*

**DOI:** 10.3390/plants11111429

**Published:** 2022-05-27

**Authors:** Idowu J. Adeosun, Itumeleng T. Baloyi, Sekelwa Cosa

**Affiliations:** Department of Biochemistry, Genetics and Microbiology, Division of Microbiology, University of Pretoria, Private Bag X20, Hatfield, Pretoria 0028, South Africa; u21747050@tuks.co.za (I.J.A.); u18372882@tuks.co.za (I.T.B.)

**Keywords:** antibacterial, exopolysaccharides, hypermucoviscosity, *Klebsiella pneumoniae*, phytochemical compounds

## Abstract

The ability of *Klebsiella pneumoniae* to form biofilm renders the pathogen recalcitrant to various antibiotics. The difficulty in managing *K. pneumoniae* related chronic infections is due to its biofilm-forming ability and associated virulence factors, necessitating the development of efficient strategies to control virulence factors. This study aimed at evaluating the inhibitory potential of selected phytochemical compounds on biofilm-associated virulence factors in *K. pneumoniae*, as well as authenticating their antibiofilm activity. Five phytochemical compounds (alpha-terpinene, camphene, fisetin, glycitein and phytol) were evaluated for their antibacterial and anti-biofilm-associated virulence factors such as exopolysaccharides, curli fibers, and hypermucoviscosity against carbapenem-resistant and extended-spectrum beta-lactamase-positive *K. pneumoniae* strains. The antibiofilm potential of these compounds was evaluated at initial cell attachment, microcolony formation and mature biofilm formation, then validated by in situ visualization using scanning electron microscopy (SEM). Exopolysaccharide surface topography was characterized using atomic force microscopy (AFM). The antibacterial activity of the compounds confirmed fisetin as the best anti-carbapenem-resistant *K. pneumoniae*, demonstrating a minimum inhibitory concentration (MIC) value of 0.0625 mg/mL. Phytol, glycitein and α-terpinene showed MIC values of 0.125 mg/mL for both strains. The assessment of the compounds for anti-virulence activity (exopolysaccharide reduction) revealed an up to 65.91% reduction in phytol and camphene. Atomic force microscopy detected marked differences between the topographies of untreated and treated (camphene and phytol) exopolysaccharides. Curli expression was inhibited at both 0.5 and 1.0 mg/mL by phytol, glycitein, fisetin and quercetin. The hypermucoviscosity was reduced by phytol, glycitein, and fisetin to the shortest mucoid string (1 mm) at 1 mg/mL. Phytol showed the highest antiadhesion activity against carbapenem-resistant and extended-spectrum beta-lactamase-positive *K. pneumoniae* (54.71% and 50.05%), respectively. Scanning electron microscopy correlated the in vitro findings, with phytol significantly altering the biofilm architecture. Phytol has antibiofilm and antivirulence potential against the highly virulent *K. pneumoniae* strains, revealing it as a potential lead compound for the management of *K. pneumoniae*-associated infections.

## 1. Introduction

Biofilms are typical forms of bacterial communities that grow on living and non-living solid surfaces, which are often immersed in a self-producing matrix [1,2]. Biofilm-associated cells can attach irreversibly to several surfaces and have become a critical worldwide health concern because of their ability to withstand antibiotics, human defense mechanisms, and other external stimuli, contributing to persistent chronic infections [3]. The adsorption of molecules to surfaces, bacterial adherence to the surface, the release of extracellular polymeric substances (EPS), microcolony formation, and biofilm maturation are all stages involved in biofilm formation [4] which have been reported in several bacteria communities.

*Klebsiella pneumoniae*, a Gram-negative bacterium belonging to the family *Enterobacteriaceae*, has been reported to form biofilms that often adhere to surfaces such as lungs, livers, and central venous catheters which are implicated in prominent nosocomial and community-acquired infections [5]. These infections include pneumonia, urinary tract infections (UTIs), bloodstream infections, necrotizing fasciitis, pyogenic liver abscess, endophthalmitis, and meningitis, among others, subsequently affecting the morbidity and mortality rate, particularly in critically ill and immunocompromised patients [6]. The interest in studying *K. pneumoniae* ATCC BAA-1705 was contingent on its ability to produce carbapenemases (KPC), which is a prevalent mechanism of resistance generated by *Klebsiella pneumoniae,* resulting in increased therapeutic dilemma and a global health threat linked to high rates of mortality [7]. Carbapenem-resistant *K. pneumoniae* is often characterized by its capacity to spread rapidly, having extensive antibiotic resistance phenotypes, yet only a few treatment options exist [7]. Furthermore, *K. pneumoniae* ATCC 700603 was also studied because it produces extended-spectrum beta-lactamases (ESBL). According to [8], the global spread of *K. pneumoniae* producing extended-spectrum lactamase (ESBL-Kp) is a serious issue; hence, the World Health Organization (WHO) categorized it alongside other ESBL-producing *Enterobacteriaceae* as a priority pathogen listed for the research and development of new antibiotics. Several antimicrobial treatments are critical in lowering the global burden imposed by this pathogen, but nonetheless, the evolution of antibiotic-resistant *K. pneumoniae* strains has become a serious public health concern [9]. *K. pneumoniae* can elude the effects of antimicrobial treatment due to the acquisition of resistance genes and the production of biofilms facilitated by EPS, making them exceedingly difficult to manage or control [10].

Antibiotic-resistant *K. pneumoniae*, which often forms biofilms, is associated with high virulence due to the nature of biofilm populations [4]. Biofilm associated virulence factors such as exopolysaccharide production, hypermucoviscosity, and the formation of curli and fimbriae also enhance the pathogenicity of this organism [11]. Bacteria protected by biofilm exopolysaccharides are up to 1000 times more resistant to antibiotics than planktonic cells [2], posing substantial therapeutic challenges and complicating treatment options.

Hypervirulent strains of *K. pneumoniae,* especially hypermucoviscous strains, also have capsule polysaccharides (CPS) for survival and immune evasion during infection, which allows them to consistently escape neutrophil-mediated intracellular killing and form abscesses at various sites, including the liver [12]. Fimbriae, another major virulence component that contributes to biofilm development in *K. pneumoniae*, consists of MrkA (capable of initiating biofilm formation) and MrkD subunits which control the binding capability and confer adhesive properties [11]. Furthermore, curli known as thin aggregative fimbriae connects directly to the substratum, which produces interbacterial bundles, allowing a cohesive and stable attachment of cells in biofilm, thereby playing an important role in biofilm development [13].

Since biofilm formation has been reported to increase virulence in *K. pneumoniae*, posing a remarkable therapeutic challenge and having developed resistance to almost all classes of conventional antibiotics [14], the development of alternative treatment options which target biofilms and related virulence factors in this pathogen is paramount.

This, therefore, necessitates the exploration of promising alternatives, which includes the search for naturally occurring compounds of plant origin capable of disrupting biofilms and their associated virulence activities. Historically, biologically active phytochemical compounds have been a valuable source of natural products, which are prominent in the prevention and treatment of diseases, helping to maintain human health [15]. The phytochemical compounds considered in this study were selected based on good docking scores and improved binding energy when bound to the SdiA protein (a transcriptional regulator which has been linked to cell division and the expression of virulence factors) in *K. pneumoniae,* as well as their drug-likeness properties which were observed during the virtual screening carried out prior to this study. The research of phytochemicals for antibacterial action, particularly against multidrug-resistant Gram-negative bacteria, has received a lot of attention in the last ten years, especially as these organisms are posing a global health challenge as well as significant economic concerns due to the rising healthcare costs [16]. This study therefore assessed the antibacterial activity and the effect of phytochemical compounds (alpha-terpinene, camphene, fisetin, glycitein and phytol) in disrupting biofilm at different stages, as well as biofilm-related virulence factors for the development of new therapeutic strategies in place of the existing conventional antibiotics. 

## 2. Results

### 2.1. In Vitro Antibacterial Validation of Selected Compounds on K. pneumoniae Strains

The antibacterial activities of five phytochemical compounds (alpha-terpinene, camphene, fisetin, glycitein and phytol) against *K. pneumoniae* strains showed minimum inhibitory concentration (MIC) values ranging from 0.0625 mg/mL to 0.250 mg/mL (Table 1). The best MIC value was shown by fisetin (0.0625 mg/mL) for *K. pneumoniae* (ATCC BAA-1705). Phytol, glycitein and alpha-terpinene showed MIC values of 0.125 mg/mL for both strains; however, camphene revealed a higher MIC value of 0.250 mg/mL. The positive controls, quercetin, a known quorum-sensing inhibitor, and ciprofloxacin, revealed significant MIC values of 0.0625 mg/mL and 0.0025 mg/mL, respectively, against both strains of *K. pneumoniae*, (Table 1). The negative control showed no inhibitory activity against both strains of *K. pneumoniae.*

### 2.2. Inhibition of K. pneumoniae Exopolysaccharides

**Figure 1:**
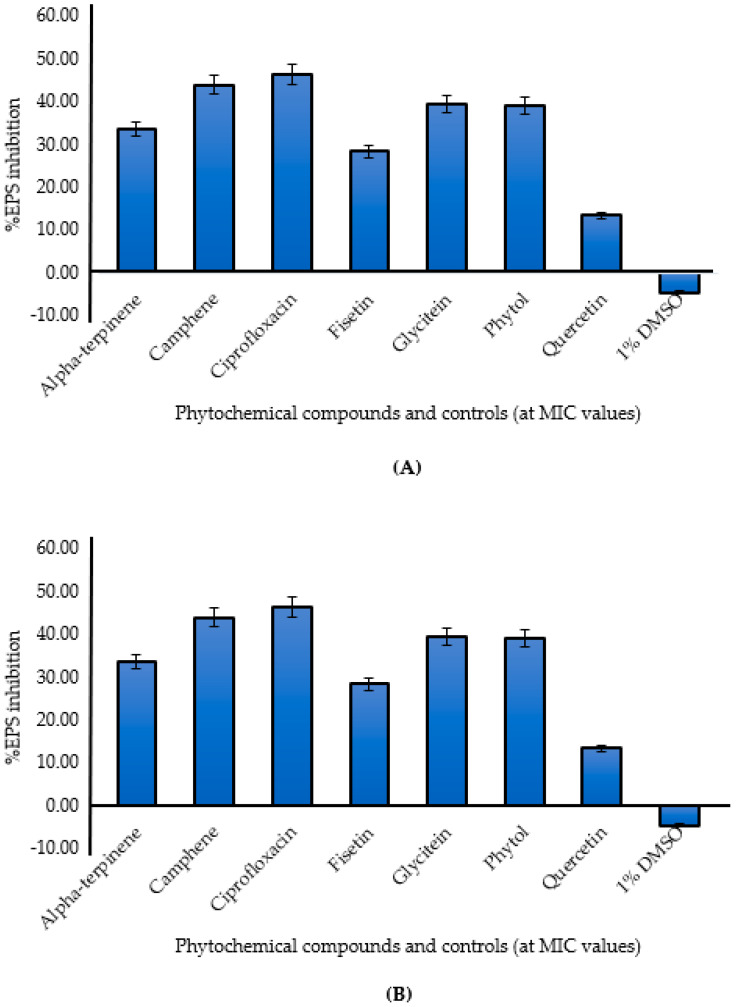
Quantification and percentage inhibition of exopolysaccharide present in *K. pneumoniae* ATCC 700603 (**A**) and *K. pneumoniae* ATCC BAA-1705 (**B**) treated with phytochemical compounds.

The quantity of EPS observed following the phenol-sulfuric acid method depicted a decrease at respective MIC values in both test pathogens. Good linearity was indicated by the correlation coefficient (R), which yielded a value of 0.971. The quantification of EPS was determined following the regression equation obtained from the standard curve Y = 0.348X − 0.074, where Y represents the absorbance obtained from the unknown samples. Figure 1 presents the EPS quantification and percentage inhibition of *K. pneumoniae* EPS by the compounds. Out of all the compounds active against the EPS production in *K. pneumoniae* ATCC 700603, the highest percentage of EPS inhibition was shown by phytol and camphene (65.91%) (Figure 1A), both having the lowest EPS yield (OD value = 1.91) (Table S1). This percentage can be compared with the result obtained for the positive control ciprofloxacin (68.45%) with a low OD value of 1.05, although the quercetin (QSI control) showed a low percentage reduction of EPS (23.21%) with an OD value of 2.68. 

Furthermore, of all the active compounds against EPS production in *K. pneumoniae* ATCC BAA-1705, camphene showed the highest percentage inhibition and/or anti-slime activity (43.80%), having the lowest EPS yield (OD value = 2.22), similar to the results observed for ciprofloxacin (EPS inhibition at 46.26% and EPS yield at 2.01 OD value) (Figure 1B). Conversely, the untreated EPS showed enhanced production for both strains of *K. pneumoniae.*

### 2.3. Microscopic Surface Topography Characterization of K. pneumoniae exopolysaccharides Using Atomic Force Microscopy 

**Figure 2:**
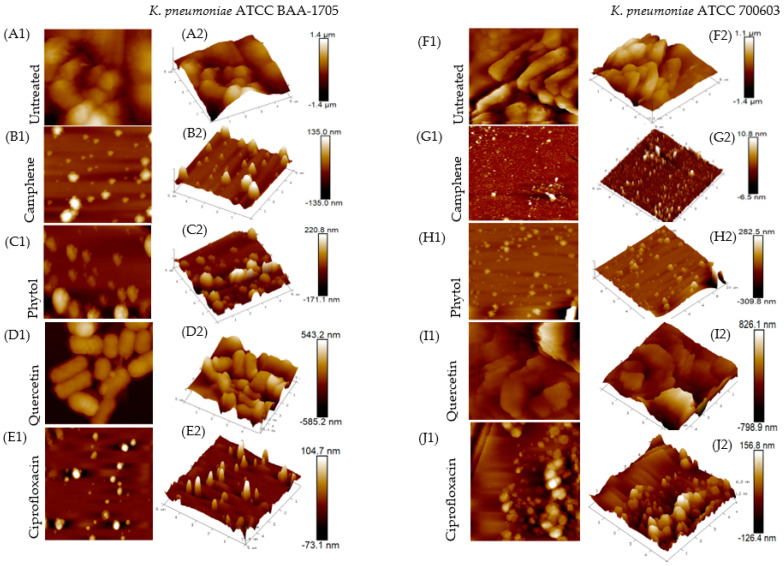
AFM images showing two-dimensional (2D) and three-dimensional (3D) surface topography of EPS produced by untreated and treated *K. pneumoniae* (ATCC BAA-1705 and ATCC 700603) strains at a scan size of 5.00 µm (5000 nm). 2D images of untreated and treated *K. pneumoniae* EPS are shown in (**A1**–**J1**). Corresponding 3D images are shown in (**A2**–**J2**).

The planar (2D) and cubic (3D) views of the surface topography of studied *K. pneumoniae* exopolysaccharides (EPS) are shown in Figure 2. AFM detected marked differences between the topographies of untreated and treated (camphene and phytol) EPS, selected due to the significant reduction in EPS. The AFM analysis revealed the irregularity and surface roughness of the EPS produced by untreated *K. pneumoniae* ATCC BAA-1705 and ATCC 700603 strains, mainly composed of unevenly distributed lumps which were clearly visible as cloudy areas around the cells (Figure 2(A1,F1)). Microscopically, the exopolysaccharides of both test strains exhibited a compact and tubular structure. Topologically, the EPS revealed a consistent polymer with a maximum height of the irregular lumps at 1.4 µm and 1.1 µm for untreated *K. pneumoniae* ATCC BAA-1705 and ATCC 700603, respectively, as shown in the 2D images (Figure 2(A1,F1)), while the average roughness (Ra) was recorded at 183 nm and 141 nm for *K. pneumoniae* ATCC BAA-1705 and ATCC 700603, respectively. The roughness parameters were obtained using the nanoscope analysis (v 8.15) software. The surface roughness is shown in the 3D images (Figure 2(A2,F2)). 

The EPS treated with camphene and phytol at the MIC value revealed visible differences in height and surface roughness in comparison with the untreated EPS. The camphene-treated EPS showed maximum lump heights of 135 nm and 10.8 nm for *K. pneumoniae*, ATCC BAA-1705 and ATCC 700603, respectively (Figure 2(B1,G1)). A significantly reduced surface roughness is shown in the 3D images (Figure 2(B2,G2)). The average roughness (Ra) was recorded at 15.6 nm for *K. pneumoniae* ATCC BAA-1705 and 1.25 nm for *K. pneumoniae* ATCC 700603. 

On the other hand, EPS treated with phytol had a maximum height of 220.8 nm and 282.5 nm for *K. pneumoniae* ATCC BAA-1705 and ATCC 700603, respectively (Figure 2(C1,H1)). The average roughness (Ra) for phytol-treated EPS was 34.8 nm for *K. pneumoniae* ATCC BAA-1705 and 25.0 nm for *K. pneumoniae* ATCC 700603. Figure 2(C2,H2) revealed a reduction in surface roughness when compared with the untreated EPS.

The EPS treated with the positive controls (quercetin and ciprofloxacin) also revealed a significant reduction in the surface roughness and height, although ciprofloxacin showed improved results with a maximum lump height at 104.7 nm and average roughness at 8.80 nm for *K. pneumoniae* ATCC BAA-1705 (Figure 2(E1,E2)). Morever, ciprofloxacin-treated *K. pneumoniae* ATCC 700603 had a maximum height of 156.8 nm and average roughness of 27.6 nm (Figure 2(J1,J2)).

### 2.4. Curli Expression Reduction in K. pneumoniae Strains by Phytochemical Compounds

The impact of the test compounds on the occurrence of curli fibers in both strains of *K. pneumoniae* is shown in Table 2. The results show that all compounds tested at 0.125 mg/mL and 0.250 mg/mL did not inhibit curli expression in the *K. pneumoniae* strains. However, phytol, glycitein, fisetin and quercetin (positive control) at concentrations of 0.5 and 1.0 mg/mL reduced the curli expression of both strains. In addition, ciprofloxacin showed a reduction in curli expression for both strains at varying concentrations (0.125 to 1.0 mg/mL) (Table 2). On the contrary, no inhibition was shown by camphene and alpha- terpinene at all concentrations. No changes were observed in bacterial colonies (which appeared red in the presence of these compounds), similarly to the negative control. 

**Figure 3:**
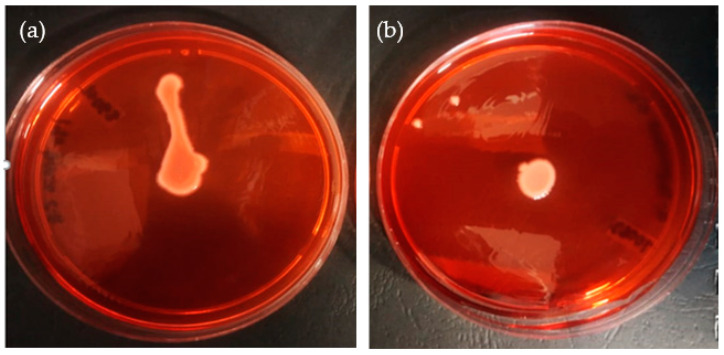
Representative images of curli expression in *K. pneumoniae*. (**a**) Negative control (untreated), showing curli-producing *K. pneumoniae*, which binds Congo red dye. (**b**) Inhibition of curli expression in *K. pneumoniae* subjected to phytol, as indicated by the appearance of white colonies on the brain–heart infusion agar plates supplemented with Congo red dye.

Figure 3 shows the representative images where no inhibition of curli expression was observed for the negative control (Figure 3a) versus observed inhibition for the phytol compound (Figure 3b). 

### 2.5. K. pneumoniae Hypermucoviscosity Reduction Using the String Test

**Figure 4:**
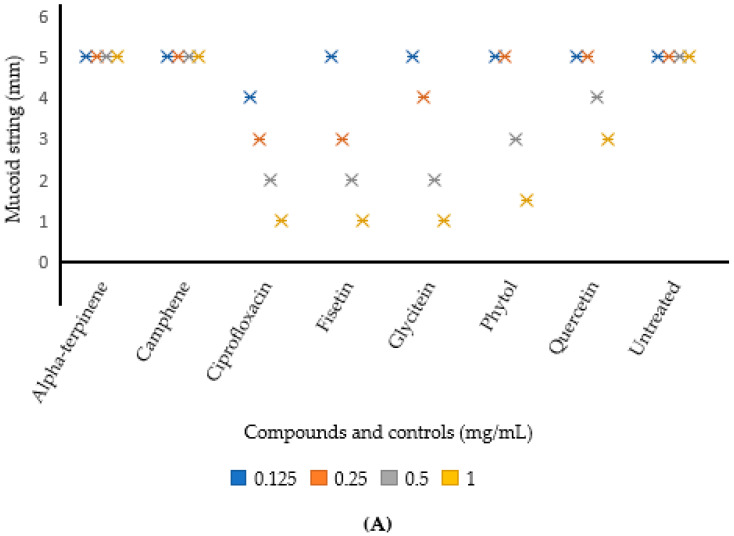

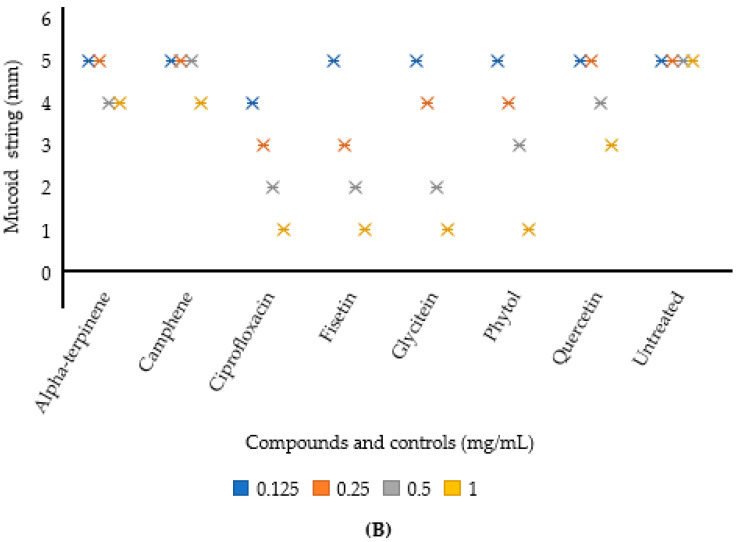
Effect of compounds on the inhibition of *K. pneumoniae* hypermucoviscosity. (**A**) For *K. pneumoniae* ATCC BAA-1705 (**B**) For *K. pneumoniae* ATCC 700603.

The effect of the test compounds on hypermucoviscosity is shown in Figure 4. The string test showed that an increase in compound concentration led to a gradual decrease in the viscosity of the test strains.

For *K. pneumoniae* ATCC BAA-1705 (Figure 4A), glycitein and fisetin revealed the potent hypermucoviscosity inhibitory activity, both showing the shortest mucoid string (1 mm) at 1.0 mg/mL (represented by the yellow *), followed by phytol (1.5 mm at 1.0 mg/mL), while no inhibitory activity was observed for camphene and alpha-terpinolene for all the concentrations tested, as seen in the negative control. Furthermore, none of the compounds showed a reduction in the hypermucoviscosity phenotype at the lowest concentration (0.125 mg/mL) tested.

Similarly, for *K. pneumoniae* ATCC 700603 (Figure 4B), glycitein, fisetin and phytol revealed potent hypermucoviscosity inhibitory activity, showing the shortest mucoid string (1 mm) at 1.0 mg/mL. However, at 0.5 mg/mL, the prominent viscosity reduction activity was shown by glycitein and fisetin, both having a mucoid string length of 2 mm. In addition, fisetin showed a potent result at the lowest concentration of 0.25 mg/mL in terms of mucoid string length reduction (3 mm), compared with the other compounds. For *K. pneumoniae* ATCC BAA-1705, no reduction in the hypermucoviscosity phenotype was shown by all the compounds at the lowest concentration tested (0.125 mg/mL), similarly to the negative control. In contrast, the positive controls (quercetin and ciprofloxacin) revealed a good reduction, with ciprofloxacin showing the highest reduction in the mucoid string length at varying concentrations for both strains (Figure 4A,B). 

### 2.6. Inhibition of Biofilm Formation

#### 2.6.1. Effect of Phytochemical Compounds on Initial Cell Attachment

The results of the anti-adhesion (initial attachment) assay against *K. pneumoniae* ATCC BAA-1705 and *K. pneumoniae* ATCC 700603 treated with test compounds are shown in Table 3. The results show that phytol had the highest inhibition of initial cell attachment for both strains tested, with 54.71% and 50.05%, respectively, followed by glycitein, which showed inhibition at 48.35% and 44.34%, respectively for both strains (Table 3). The least anti-adhesion activity was shown by camphene at 22.27% for *K. pneumoniae* ATCC BAA-1705 and 18.53% for *K. pneumoniae* ATCC 700603. Quercetin and ciprofloxacin revealed an initial cell attachment inhibition at 42.57% and 69.25% for *K. pneumoniae* ATCC BAA-1705, while for *K. pneumoniae* ATCC 700603, 40.66% and 62.45% were observed for quercetin and ciprofloxacin, respectively. No inhibition was revealed by the negative control (Table 3). A statistically significant difference was observed between phytol and the other compounds tested (ANOVA GLM, F = 14.14, DF = 5, R2 = 0.049, *p* < 0.05). Phytol showed potent activity since it revealed >50% inhibition, while glycitein, camphene, fisetin and alpha-terpinene showed weak activity, having percentage inhibition values between 0 and 49%.

#### 2.6.2. Effect of Phytochemical Compounds on Preformed Biofilm Inhibition: Biomass Measurement

The inhibition of biofilm microcolonies formed by the test strains upon treatment with the phytochemical compounds was assessed, and the results are shown in Table 3. The percentage inhibition of preformed biofilm by the compounds was observed to be slightly less compared with the initial cell attachment, with the highest biofilm reduction of 43.81% shown by phytol for *K. pneumoniae* ATCC BAA-1705. Glycitein also revealed 39.61% inhibition for *K. pneumoniae* ATCC BAA-1705 and 32.77% inhibition for *K. pneumoniae ATCC* 700603, which is slightly higher than the results obtained for quercetin, showing 35.15% and 31.81% inhibition for *K. pneumoniae* ATCC BAA-1705 and *K. pneumoniae ATCC* 700603, respectively. However, the highest percentage inhibition was observed for ciprofloxacin at 56.42% and 51.77% for *K. pneumoniae* ATCC BAA-1705 and *K. pneumoniae ATCC* 700603, respectively (Table 3). The negative control did not reveal any inhibitory effect on the biofilm development, except for a slightly enhanced biofilm formation.

#### 2.6.3. Disruption of Mature Biofilm by Phytochemical Compounds 

Mature biofilms formed by *K. pneumoniae* strains determined under dynamic and static conditions are shown in Table 4. The highest disruption of mature biofilms under dynamic conditions was shown by phytol at 24.94% and 25.88% for *K. pneumoniae* (ATCC BAA-1705) and *K. pneumoniae* (ATCC 700603), respectively. The least inhibition was shown by camphene at 5.24% for *K. pneumoniae* (ATCC BAA-1705) and 2.06% for *K. pneumoniae* (ATCC 700603). 

Under static conditions, phytol again revealed higher inhibitory activity (20.32% and 18.07% for both strains), followed by alpha-terpinene (Table 4), with statistical differences found between the compounds (ANOVA GLM, F = 25.84, DF = 5, R2 = 0.0139, *p* < 0.05). The least inhibitory activity for this group was also shown by camphene at 4.56% for *K. pneumoniae* (ATCC BAA-1705) and 4.08% for *K. pneumoniae* (ATCC 700603). Moreover, fisetin and quercetin showed no inhibitory activity on mature biofilms formed by both strains under static conditions. 

Overall, less inhibitory activity was shown by the compounds on mature biofilms under static conditions compared with dynamic conditions. This was also observed for the positive control of ciprofloxacin (Table 4). 

### 2.7. In Situ Visualisation of Biofilms Using Scanning Electron Microscopy 

**Figure 5:**
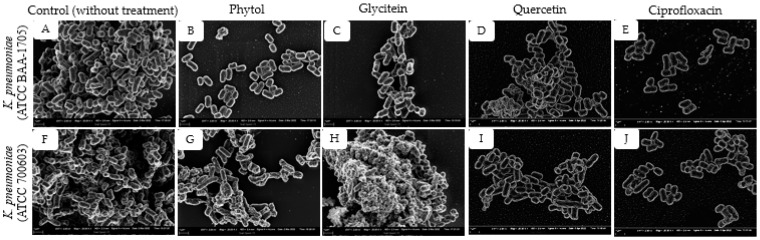
SEM micrographs showing the biofilm inhibitory activity of phytol and glycitein against *K. pneumoniae* ATCC BAA-1705 and ATCC 700603 at ×20,000 magnification. (**A**) *K. pneumoniae* ATCC BAA-1705 (without treatment). (**B**) *K. pneumoniae* ATCC BAA-1705 (treated with phytol). (**C**) *K. pneumoniae* ATCC BAA-1705 (treated with glycitein). (**D**) *K. pneumoniae* ATCC BAA-1705 (treated with quercetin). (**E**) *K. pneumoniae* ATCC BAA-1705 (treated with ciprofloxacin). (**F**) *K. pneumoniae* ATCC 700603 (without treatment). (**G**) *K. pneumoniae* ATCC 700603 (treated with phytol). (**H**) *K. pneumoniae* ATCC 700603 (treated with glycitein). (**I**) *K. pneumoniae* ATCC 700603 (treated with quercetin). (**J**) *K. pneumoniae* ATCC 700603 (treated with ciprofloxacin).

To further investigate the detailed effects of the *K. pneumoniae* biofilms formed after treatment with the best active or potent compounds (phytol and glycitein) on antibiofilm assay results, a scanning electron microscope analysis was carried out. Figure 5 shows the SEM micrographs of the biofilms formed by the two *K. pneumoniae* strains upon exposure to phytol and glycitein (0.1 mg/mL), the positive controls (quercetin; 0.1 mg/mL and ciprofloxacin; 0.001 mg/mL), and the untreated biofilms.

Phytol revealed potent antibiofilm activity for *K. pneumoniae* (ATCC BAA-1705) and *K. pneumoniae* (ATCC 700603), as evidenced in Figure 5B,G, where fewer clumps of attached microcolonies were observed, revealing a notable lessening in the quantity of biofilms with some of the cells being distances apart. A similar observation was recorded for ciprofloxacin, showing very few clumps of scattered cells (Figure 5E,J). 

In comparison, the untreated biofilms formed by the two strains of *K. pneumoniae* revealed a compact arrangement of interconnected *K. pneumoniae* cells, thereby presenting continuous clumps and large aggregates of cells (Figure 5A,F). However, biofilms treated with glycitein only revealed a slight distance amongst the cells of *K. pneumoniae* (ATCC BAA-1705) (Figure 5C) while the treatment was shown to shrink and disrupt cells of *K. pneumoniae* (ATCC 700603) with extruding materials and cell debris (Figure 5H). Quercetin was less effective compared with phytol and glycitein (Figure 5D,I); moreover, it showed lesser clumps of cells than the untreated biofilms.

## 3. Discussion

Plants have been known for their antibacterial effects against microbial pathogens since ancient times due to their secondary metabolites [17,18]. Phytochemicals have vast advantages over synthetic compounds, including green status and unique modes of action, which could aid in the fight against antibiotic resistance. Hence, phytochemicals are emphasized as a valuable source of novel bioactive compounds that is both sustainable and abundant [18]. Furthermore, they have been identified as a promising source of quorum-sensing inhibitors, disrupting bacterial cell-to-cell communication, which enables pathogenicity and for bacteria to withstand antimicrobial substances through biofilm formation and other virulence factors [19]. These phytochemicals often have a wide range of chemical variety, structural complexity, and biological activity [19], making them promising tools for the management of illnesses, especially biofilm-related infections in an era where the supply of effective antibiotics is no longer guaranteed [20]. New sources of antimicrobials and tactics for effective biofilm inhibition and/or eradication are unquestionably necessary. Therefore, the discovery of phytochemicals targeting distinct stages of biofilm formation, such as adhesion, motility and EPS generation, including other biofilm-related virulence factors, is imperative [16]. As such, this study explored the effects of phytochemical compounds at different stages of biofilm formation and associated virulence factors in *K. pneumoniae* ATCC BAA-1705 and *K. pneumoniae* ATCC 700603. 

The five studied compounds (alpha-terpinene, camphene, fisetin, glycitein and phytol) were first validated for their antibacterial effect on the growth of *K. pneumoniae* ATCC BAA-1705 and *K. pneumoniae* ATCC 700603 strains. The findings reveal MIC values ranging from 0.0625 to 0.25 mg/mL (Table 1). Of the five compounds, fisetin showed anMIC value of 0.0625 for *K. pneumoniae* (ATCC BAA-1705), as well as quercetin and ciprofloxacin (the positive controls), which indicated significant activity. This suggests that fisetin and quercetin are potential antibacterial agents against the studied pathogen. Their potent activity can be attributed to the mode of action of flavonoids, which includes the interaction of phytochemical compounds with bacterial proteins and cell wall structures [21]. This is congruent with the suggestions of Gibbons [22] and Mamabolo [23], where the antimicrobial activity of a phytochemical compound or single entity compound is defined as significant when the MIC value is ≤ 0.064mg/mL or ≤ 0.01mg/mL, respectively. 

Other tested compounds revealed MIC values greater than 0.1 mg/mL; hence, they are regarded as compounds with low antibacterial activity. According to Mbaveng et al. [24], the MIC activity of a compound is considered low when it is greater than 100 µg/mL or 0.1mg/mL. The low MIC values obtained may be due to the protective outer membrane present in *K. pneumoniae*, being a Gram-negative bacterium [25]. In addition, they can also be attributed to the ability of *K. pneumoniae* to actively efflux the compounds from the cell, forming a capsule that shields the cell from being penetrated by the compounds or changing its phytocompound target. The low MIC values, however, do not completely rule out the bioactive potentials of these compounds as they possess a broad range of biological activities. Cosa et al. [26] and Vasavi et al. [27] reported that in some cases, compounds of natural origin may yield poor MIC values, but they are able to interfere with the quorum-sensing signaling mechanism and inhibit virulence at sub-MIC concentrations. Hence, the compounds were further assessed for their biofilm-associated anti-virulence activities.

Because biofilms are supported by a matrix of polymeric compounds known as extracellular polymeric substances (EPS), often composed of exopolysaccharides that are secreted into the environment [28], we assessed this as one of the contributing virulence factors. *Klebsiella pneumoniae*’s exopolysaccharides generally contain rare sugars such as L-fucose, L-rhamnose, or uronic acids [29,30]. Based on our findings, the exopolysaccharide reduction assay revealed that both phytol and camphene showed the highest percentage inhibition of EPS (65.91%) for *K. pneumoniae* ATCC 700603, while camphene revealed the greatest reduction in exopolysaccharide production (43.80%) in *K. pneumoniae* ATCC BAA-1705 (Figure 1). 

Similar findings were reported by Srinivasan et al. [31], where phytol significantly inhibited the EPS production in *Serratia marcescens* to the level of 32% and 39% at 5 and 10 µg/mL concentrations, respectively, while no significant level of EPS inhibition was shown by the control. The bioactive potential of camphene observed in this study is congruent with the submission of Hachlafi et al. [32], where camphene inhibited pathogenicity in a wide range of pathogenic bacteria, such as *Klebsiella pneumoniae, Staphylococcus aureus* and *Escherichia coli.* Based on our findings, the reduction in EPS in *K. pneumoniae* by the active phytochemical compounds suggests their potential to disrupt biofilm-associated virulence factors. This is because EPS production is a key factor which forms the framework in microbial biofilms.

The validation of reduced exopolysaccharides in *K. pneumoniae* was performed using atomic force microscopy (AFM), a powerful technique for imaging the surfaces of microbial cells [33]. It has been reported as a vital tool in characterizing the topographic features of microbial exopolysaccharides [34]. Dufrene [35] also confirmed that AFM imaging allows the observation of cell wall components directly on live cells, such as polysaccharides, peptidoglycan, teichoic acids, among others, and has aided in elucidating their roles in cellular processes such as adhesion. When AFM imaging was employed in our study to analyze the surface topology of treated and untreated *K. pneumoniae* exopolysaccharides, the untreated strains resulted in the formation of a clear detectable EPS network composed of unevenly distributed and compact lumps (Figure 2(A1,F1)). The lumps may be formed due to the intra- and intermolecular aggregation of polysaccharide macromolecules [36]. This high conformational rigidity of EPS might function as a polymeric scaffold used by bacteria to build biofilms [37]. The surface topography of EPS treated with phytol and camphene on the other hand revealed scarce EPS polymers which were generally thinner and often showed irregular shapes, similar to the positive control (ciprofloxacin) (Figure 2(B1,C1,G1,H1)). These compounds showed a significant reduction in the height and surface roughness of *K. pneumoniae* EPS. This validates the results obtained from the phenol sulfuric acid method of EPS biomass measurement.

Curli, a type of fimbriae composed of proteins called curlins and functional amyloid surface fiber, is another prominent virulence factor in *K. pneumoniae* known to be involved in cell attachment to surfaces, as well as cell aggregation, which allows the formation of biofilms [38]. Curli are effective inducers of the host inflammatory response and often mediate host cell adhesion and invasion [39]. Our results clearly demonstrate that phytochemicals such as phytol, glycitein, fisetin and quercetin (0.5 and 1.0 mg/mL) efficiently inhibited the formation of curli in the *K. pneumoniae* strains (Table 2). According to a study by Gupta et al. [40], cranberry, which contains diverse bioactive phytochemical compounds, inhibited the expression of curli in *Escherichia coli* and resulted in a loss of epithelial cell colonization. This suggests that certain phytochemical compounds can bind to curli, and fimbriae as observed in our study, thereby preventing them from attaching to host tissue. According to Kikuchi et al. [13], studies have shown that curli and other cell surface structures play a significant role in the development of biofilm in *E. coli*, an *Enterobacteriaceae* similar to *K. pneumoniae*. Understanding and inhibiting biofilm-forming structures such as curli are crucial for the development of therapeutics that can reduce biofilm formation and host colonization [41].

Furthermore, virulence in *K. pneumoniae* can also be attributed to efficient iron uptake, poor sedimentation and the copious synthesis of a capsule, which confers a hypermucoviscous phenotype [42,43]. The effect of the studied phytochemical compounds on the hypermucoviscosity of *K. pneumoniae* was examined using the string test. The results reveal glycitein and fisetin as the compounds showing the best inhibition at 1.0 mg/mL for both strains, alongside phytol for *K. pneumoniae* ATCC 700603 (Figure 4). The viscosity-lowering effect of the compounds, as seen in this study, is proportional to their concentrations, as none of the compounds examined at the lowest concentration (0.125 mg/mL) showed any reduction in the hypermucoviscosity phenotype. A similar observation was also recorded in the study of Jabuk [44], where viscosity inhibition was observed in a dose-dependent manner. Lin et al. [45] reported a decrease in *K. pneumoniae* mucoviscosity, and capsular polysaccharide production by *Fructus mume* in a dose-dependent manner, thereby reducing the resistance of *K. pneumoniae* to serum killing.

*K. pneumoniae* can produce a thick extracellular matrix that promotes bacterial adhesion to living or non-living surfaces, preventing antibiotic penetration and lowering the effects of treatments [46]. Again, the host defenses may be improved if any stage in the formation of biofilm’s structure is interrupted, resulting in better treatment outcomes. Hence, this study examined the effect of phytochemical compounds on initial cell attachment, preformed biofilm, and mature biofilm formation. 

The results of the initial cell attachment inhibition reveal that phytol showed good activity on both strains of *K. pneumoniae* tested following the criteria stated by Famuyide et al. [47], having >50% inhibition (Table 3). Reports on the anti-adhesion activity of the studied compounds on *K. pneumoniae* are limited; however, Ramanathan et al. [48] reported a good anti-biofilm activity of phytol, showing up to 60% biofilm inhibition in another notorious biofilm former, *Acinetobacter baumannii,* at concentrations ranging from 5 to 640 µg/mL. Congruent to our findings, Srinivisan et al. [31] also reported a decrease in the level of metabolically active cells involved in biofilm formation in phytol treatment compared with their respective controls. This corroborates the submission of Ramanathan et al. [48], that phytol is a potential anti-biofilm agent, as it can inhibit or halt the formation of biofilms, making them more receptive to treatments. On the other hand, glycitein, camphene, fisetin, alpha-terpinene and quercetin showed weak anti-adhesion activity, having percentage inhibition values <49%. The weak activity observed might be attributed to the interference of the hydrogen bonds, electrostatic forces, and van der Waals forces of interaction within the biofilm, which often mediates the initial attachment of the sessile group of cells to solid surfaces [21]. 

Furthermore, our results reveal a reduced inhibition of the microcolony formation stage by the compounds (Table 3). This suggests that biofilms can be better inhibited during the initial cell attachment stage than when they begin to develop. A similar trend was observed for the inhibition of mature biofilm, where the biofilms had accumulated biomass. These findings are in tandem with results obtained in a study carried out by Mombeshora et al. [5], where the compound tested did not have any disruptive effect on mature (72 h) biofilms of *P. aeruginosa*, a Gram-negative bacterium like *K. pneumoniae*. This can also be attributed to the opinion of Kelmanson et al. [49], who noted that more resistance to external agents is often shown once biofilms have been fully established; therefore, the disruption of mature biofilms tends to require higher doses of disrupting agents than those needed to destroy planktonic cells. Additionally, difficulty in the disruption of mature biofilms might result from the slow or incomplete penetration of the treatments to the established biofilm population or an altered biochemical microenvironment within the biofilm [50]. Other studies by Baloyi et al. [51] and Sarkar et al. [52] have also shown that eradicating biofilms is challenging, as various biofilm-forming microorganisms have demonstrated resilience.

The effect of the phytochemical compounds on the inhibition of mature *K. pneumoniae* biofilm was assessed under both static and dynamic conditions. The results revealed that *K. pneumoniae* biofilms formed under static (non-shaking) conditions had lower inhibition percentages compared with the mature biofilm formed under dynamic conditions (Table 4). This could be because more mature biofilms were formed without shaking compared with the biofilms formed while shaking; hence, the treatment showed higher inhibitory activity on less mature biofilms formed while shaking. This result corroborates the findings of [53], where a decrease was observed in the biofilm biomass attached to substratum surfaces under dynamic conditions compared with the static condition. The difference in mature biofilms generated with and without shaking can be attributed to shear force, which is one of the most decisive factors in the formation of biofilms in hydrodynamic conditions [53]. Due to shear forces, bacteria that settled but could not adhere securely to the substratum surface might have been resuspended in the bulk liquid, resulting in relatively low levels of adherent biomass under dynamic conditions. Bacterial adhesion, which contributes to mature biofilm formation, is often inhibited when there is an increase in shear stress [54].

An additional remarkable mechanism in biofilm formation is the distinctive biofilm architecture [48]. SEM micrographs of the structurally complex matrix architecture and the bacteria in that matrix were used to visually validate the inhibitory effect of phytol and glycitein against biofilms formed by the two *K. pneumoniae* strains. Exceptionally, phytol treatment led to a huge collapse in the extracellular matrix architecture of *K. pneumoniae* (ATCC BAA-1705) biofilms, resulting in individual cells and loose microcolonies adhered to the coverslip (Figure 5B). The images correlated well with the quantitative results of the crystal violet staining assay, which indicated that phytol possessed good antibiofilm activity against *K. pneumoniae*. Furthermore, glycitein influenced the integrity of the *K. pneumoniae* (ATCC 700603) cell wall (Figure 5F), making the cells incapable of maintaining their typical morphology in the presence of the treatment. Damaged cell walls and cellular leakages resulting from phytochemical compound treatment can eventually cause the death of microbial cells [55].

The inhibition of the biofilm-forming ability and associated virulence factors in *K. pneumoniae* by selected phytochemical compounds could be an effective approach in controlling pathogenicity in this pathogen.

## 4. Materials and Methods

### 4.1. Chemicals, Media and Compounds Used in Assays 

Chemicals used in the study, including dimethyl sulfoxide (DMSO), iodonitrotetrazolium (INT), hexamethyldisilazane (HMDS), Congo red and crystal violet were purchased from Sigma-Aldrich (Johannesburg, South Africa). Luria Bertani agar (LBA), Luria Bertani broth (LBB), brain–heart infusion agar (BHIA), blood agar (BA) and Muller–Hinton broth (MHB) were purchased from Lasec (Johannesburg, South Africa). Compounds and positive controls used in the study, including phytol (lot no: 0001452396), glycitein (lot no: MFCD00016679), camphene (lot no: MKCL4074), fisetin (lot no: 82542), alpha-terpinene (lot no: BCCD2529), quercetin (lot no: LRAB7760) and ciprofloxacin (lot no: 098M4006V), were purchased from Sigma-Aldrich (Johannesburg, South Africa). 

### 4.2. Bacterial Strains and Growth Conditions 

American Type Culture Collection strains of *K. pneumoniae* (ATCC BAA-1705 and ATCC 700603) were obtained from the NextGen Health unit at the Council for Scientific and Industrial Research (CSIR), South Africa. The bacterial strains were kept as glycerol stocks at −80 °C until required for use. The two strains of *K. pneumoniae* used in this study were prepared in Mueller–Hinton (MH) medium during MIC assay and incubated at 37 °C to obtain active bacterial cultures. One or two colonies were often transferred to sterile distilled water to obtain an absorbance (OD_600 nm_) of 0.1. The cell suspension was adjusted to achieve a 0.5 McFarland standard equivalent.

Ethics approval for the use of the *K. pneumoniae* strains was sought and obtained from the University of Pretoria, Faculty of Natural and Agricultural Sciences Ethics Committee (reference number: NAS157/2021).

### 4.3. Antibacterial Activity of Phytochemical Compounds against K. pneumoniae Strains

The MIC of the phytochemical compounds was determined following the broth dilution method as described by Alves et al. [56], with slight modifications. Stock concentrations (1 mg/mL) of the compounds were prepared and 100 µL of MH broth was transferred into each well of a 96-well microtiter plate. A 100 µL aliquot of each phytochemical compound (in triplicate) was transferred into the first row of microtiter plates. Serial dilutions were performed in the direction from A to H, resulting in decreasing concentrations over the range of 0.25–0.0019 mg/mL. Subsequently, 100 µL of the standardized bacterial strains (OD _600 nm_ = 0.08–0.1) was transferred into each well. Each plate was prepared with a set of positive and negative controls. Quercetin and ciprofloxacin were used as the positive controls at a concentration of 1 mg/mL and 0.01 mg/mL, respectively, while 100 µL of 1% DMSO was used as the negative control. Following incubation at 37 °C for 24 h, 40 µL of a 0.20 mg/mL solution of p-iodonitrotetrazolium violet (INT) was added to each well and incubated at 37 °C for 30 min. The MIC value for each phytochemical compound was visually assessed and recorded. The MIC was recorded as the minimum concentration of the compounds at which there was no visible growth of the test strain. The antibacterial assay was carried out in triplicate.

### 4.4. Inhibition of Biofilm-Associated Virulence Factor—Exopolysaccharide Assay

Reduction in EPS was carried out according to the method described by Gopu and Shetty [57]. A sterile LB broth with and without the active compound(s) was inoculated with 1% *Klebsiella pneumoniae* and incubated at 37 °C. Biofilms that adhered to the walls of the test tubes were harvested to obtain crude EPS. Briefly, late log phase cells were removed by centrifugation at 5000× *g* for 30 min at 2 °C. The filtered supernatant was added to three volumes of chilled ethanol and incubated overnight at 2 °C to precipitate the dislodged EPS. Precipitated EPS was collected by centrifugation at 8000×*g* for 30 min then dissolved in 1 mL of deionized water and stored at −40 °C until further use. EPS was quantified by mixing 1 mL of EPS solution with an equal volume of 5% phenol and 5 mL of concentrated sulfuric acid to develop a red color. Glucose with a concentration range between 0.25 and 1 mg/mL was used as a standard to determine the R^2^ value in the calibration and for the quantification of crude EPS. The intensity of the color developed was measured using a microplate reader (Biotek, United States of America) at 490 nm.

### 4.5. Assessment of Exopolysaccharide Inhibition Using Atomic Force Microscopy 

The effect of the best two compounds (phytol and camphene) shown to reveal noteworthy exopolysaccharide inhibition in *K. pneumoniae* strains was monitored using atomic force microscopy following the method described by Santana et al. [58]. Two *K. pneumoniae* strains (ATCC BAA-1705 and ATCC 700603) were grown overnight in LB media, centrifuged (2000× *g*, room temperature, 15 min), washed three times in phosphate buffer (5 mM, pH 6.5), and approximately 10^8^ CFU ml-1 were resuspended into tubes containing the same buffer. The phytochemical compounds were diluted to 1 mg/mL and 100 µL of compounds were added to 3 mL of the cell suspensions. The samples were incubated for 4 h at 37 °C. Cell suspensions without the addition of the compounds were used as controls. 

After incubation, samples of 1 mL were collected from each treatment, centrifuged (6000×*g*, at room temperature for 15 min) and a smear of cells was prepared in a glass slide (191 cm). The slides were air dried and viewed using the atomic force microscope at the Microscopy Unit, University of Pretoria, South Africa. 

Samples were observed in a contact imaging mode using a Veeco atomic force microscope (Dimension icon with Scan Asyst) and silicon tip on nitride lever (cantilever 0.55–0.75 µm). A nominal constant of 32 Nm-1 and resonance frequency of ≈300 kHz was used with a scan rate of 0.100 Hz and scan size of 5.00 µm. Imaging analysis was performed using the Nanoscope analysis Scan Asyst software (Nanoscope version 8.15).

### 4.6. Inhibition of Curli Expression

The effect of five test compounds on curli expression was assessed according to the method described by Jabuk [44] with slight modifications. The bacterial suspension was prepared by inoculating 100 µL of standardized *K. pneumoniae* strains and 100 µL of the compounds in 3 mL of LB broth. The suspension was incubated at 37 °C for 24 h. After incubation, 3 µL of the bacterial suspension was inoculated onto plates containing brain–heart infusion (BHI) agar supplemented with Congo red (CRI) dye and sucrose. Curli-producing *K. pneumoniae* bound to Congo red dye and formed red colonies, whereas curli-negative bacteria formed white colonies, which indicated a loss of curli fimbriae. Control cultures contained no compounds. 

### 4.7. Reduction in Hypermucoviscosity Using the String Test

A variation of the string test was used to determine the effect of the studied compounds on the hypermucoviscousity (HMV) phenotype of *K. pneumoniae* strains according to the method described by Wiskur et al. [59] and Jabuk [44]. K. pneumoniae strains were inoculated on BHI plates containing the five phytochemical compounds with varying concentrations (0.125–1.0 mg/mL) and incubated overnight at 37 °C. A standard bacteriological loop was used to vertically stretch a mucoviscous string from a single colony. Each strain was defined as mucoid or regarded as a hypermucoviscous (HMV+) phenotype when string-like growth or a mucoid string of >5 mm was observed, respectively. The control culture contained no compounds.

### 4.8. Effect of Phytochemical Compounds on Biofilm Formation—Initial Cell Attachment, Preformed Biofilm and Mature Biofilm

Anti-adhesion (initial cell attachment), preformed biofilm (biomass measurement) and mature biofilms were assessed for inhibition by the phytochemical compounds, following the method described by Baloyi et al. [51] and Blando et al. [60], with slight modifications. 

Five phytochemical compounds (alpha-terpinene, camphene, fisetin, glycitein and phytol) were tested against *K. pneumoniae strains* (ATCC BAA-1705 and ATCC 700603) for initial cell attachment, preformed and mature biofilm inhibition. For the initial cell attachment inhibition assay, 100 µL of standardized bacterial suspension (OD_600 nm_ = 0.1), 100 µL of MH broth and 100 µL of the compound were added to the wells. The positive controls (quercetin 0.1 mg/mL and ciprofloxacin, 0.001 mg/mL) and negative control (1% DMSO) were also added into the wells, which were then incubated at 37 °C for 24 h.

For preformed and mature biofilm assays, 100 µL of standardized bacterial suspension and 100 µL of MH broth were added to the wells and incubated at 37 °C for 8 h for preformed biofilm, while for mature biofilm, wells were incubated at 37 °C with and without shaking for 24 h. Following incubation, 100 µL of the compounds was transferred to individual wells and incubated for another 24 h. The modified crystal violet (CV) assay was used to analyze initial cell attachment, biofilm biomass, and mature biofilms. To eliminate planktonic cells and medium, the 96-well plates containing developed biofilm were rinsed with sterile distilled water. Afterwards, the plates were dried in the oven for 45 min at 60 °C. After drying, the remaining biofilm was stained for 15 min in the dark with a 1% CV solution (Sigma-Aldrich, Johannesburg, South Africa). To eliminate any unabsorbed stain, the wells were washed with sterile distilled water. Destaining the wells with 125 µL of 95% ethanol allowed for the semiquantitative measurement of biofilm formation. Approximately 100 µL of the destaining solution was transferred to a new plate and the absorbance (OD_585 nm_) was read using a multi-mode microplate reader (SpectraMax^®^ paradigm). The % inhibition of test compounds was calculated from the untreated broth culture. The formula below was used in calculating the percentage of inhibition:

$$\mathrm{Biofilm}\;\mathrm{reduction}\;\left(\%\right)=({\mathrm{Control}}_{585\mathrm{nm}}-{\mathrm{Test}}_{585\mathrm{nm}})/\left({\mathrm{Control}}_{585\mathrm{nm}}\right)\times 100$$

Results were interpreted following the criterion described by Famuyide et al. [47]. Values between 0 and 100% were interpreted as inhibitory activity; however, it was further broken down as follows: ≥ 50% was interpreted as good activity, and values between 0 and 49% were interpreted as weak activity, while negative values indicated a growth increase rather than the inhibition of biofilm.

### 4.9. In Situ Visualization of Biofilms Using Scanning Electron Microscopy 

Subinhibitory biofilm inhibitory concentrations of the two most active compounds (phytol and glycitein) were fixed and visualized in a field emission gun scanning electron microscope to observe the cell density and morphology of biofilms following the method described by Wijesundara and Rupasinghe [61] with slight modifications. Biofilms were fixed (while still in a microtiter plate) over a minimum of 2 h in 0.1 M sodium cacodylate buffer (pH 7.2) containing 2% glutaraldehyde immediately after being rinsed in PBS. The biofilms were further rinsed three times with phosphate washing buffer 3 times for 15 min each. Then, the samples were dehydrated through an ethanol gradient series (35%, 50%, 75%, 90% and 100%). All the steps in the gradient involved 15 min exposure times, with the final 100% ethanol treatment being repeated three times. Drying of samples was achieved through an ethanol gradient series (25:75, 50:50, 75:25 and 100:0) for 15 min at each concentration. The 100:0 dilution step was repeated three times. A 50:50 mixture of hexamethyldisilazane (HMDS) and 100% ethanol was added and allowed to stay for 1 h with the samples covered. The HMDS–ethanol mixture was removed and fresh HMDS was added. Plates were air-dried under the fume hood for 2 h. Finally, fixed biofilms were mounted on aluminum stubs. Then, sputters were coated with gold–palladium (15 nm) and visualized using a Zeiss crossbeam 540 scanning electron microscope with operational conditions of an acceleration voltage of 10 kV, emission current of 14–16 μA, working distance of 10–12 mm and analysis lens mode.

### 4.10. Statistical Analysis

All results were presented as mean *±* standard deviations for each sample and treatment. Data were generated in independent experimental repeats with each sample in triplicates. The ANOVA generalized linear model (Proc GLM) was used to analyze the means of inhibitory activities of the compounds and controls. All statistical analyses were carried out using the Statistical Analysis System (SAS) program version 9.4 from Stats Inc., 100 SAS Campus Drive, Cary, NC, USA, with *p* < 0.05 values considered statistically significant.

## 5. Conclusions

In this study, a better knowledge of the efficacy of selected phytochemical compounds was acquired by investigating antivirulence and antibiofilm activities. AFM proved to be a useful tool for visualizing the effect of compounds on EPS production in order to corroborate the in vitro findings. Amongst the phytochemical compounds evaluated in this study, phytol proved to be the most potent antivirulence antibiofilm agent, inhibiting initial cell attachment as well as exopolysaccharide production, curli expression and hypermucoviscosity. Consequently, this intriguing compound can be employed as a model in the search for new medications or as an alternative in regulating the pathogenicity of *K. pneumoniae*. 

## Figures and Tables

**Table 1:** MIC values of selected phytochemical compounds on *K. pneumoniae* strains.

Compounds	*K. pneumoniae* Strains and MIC (mg/mL) Values	
	*K. pneumoniae* (ATCC BAA-1705)	*K. pneumoniae* (ATCC 700603)
Alpha-terpinene	0.125	0.125
Camphene	0.250	0.250
Fisetin	0.0625	0.125
Glycitein	0.125	0.125
Phytol	0.125	0.125
**Controls**		
Ciprofloxacin	0.0025	0.0025
Quercetin	0.0625	0.0625

The MIC values are presented as the mean values of triplicates.

**Table 2:** Effect of compounds on curli fiber synthesis in *Klebsiella pneumoniae* strains.

Compounds	Concentration (mg/mL) (A)					Concentration (mg/mL) (B)				
	Control	0.125	0.250	0.5	1.0	Control	0.125	0.250	0.5	1.0
Alpha-terpinene	+	+	+	+	+	+	+	+	+	+
Camphene	+	+	+	+	+	+	+	+	+	+
Fisetin	+	+	+	-	-	+	+	+	-	-
Glycitein	+	+	+	-	-	+	+	+	-	-
Phytol	+	+	+	-	-	+	+	+	-	-
**Controls**										
Ciprofloxacin	+	-	-	-	-	+	-	-	-	-
Quercetin	+	+	+	-	-	+	+	+	-	-
Untreated	+	+	+	+	+	+	+	+	+	+

Key: + (Present), - (Absent), A = ATCC BAA-1705, B = ATCC 700603.

**Table 3:** Effect of phytochemical compounds on initial cell attachment (anti-adhesion) and biofilm development of *K. pneumoniae* strains.

Compounds	Percentage (%) Inhibition of Initial Cell Attachment		Percentage (%) Inhibition of Biofilm Development	
	*K. pneumoniae* (ATCC BAA-1705)	*K. pneumoniae* (ATCC 700603)	*K. pneumoniae* (ATCC BAA-1705)	*K. pneumoniae* (ATCC 700603)
Alpha-terpinene	33.71 ± 0.01 ^a,b^	37.05 ± 0.00 ^a,b^	17.23 ± 0.04 ^b,c^	19.04 ± 0.03 ^a,b^
Camphene	22.27 ± 0.08 ^a^	18.53 ± 0.01 ^a^	14.58 ± 0.04 ^a^	11.08 ± 0.02 ^a^
Fisetin	39.81 ± 0.01 ^a,b^	32.59 ± 0.04 ^a^	25.79 ± 0.00 ^a,b^	29.93 ± 0.02 ^a,b^
Glycitein	48.35 ± 0.02 ^b,c^	44.34 ± 0.02 ^c^	39.61 ± 0.01 ^d^	32.77 ± 0.04 ^b^
Phytol	54.71 ± 0.01 ^c^	50.05 ± 0.00 ^c^	43.81 ± 0.01 ^e^	40.02 ± 0.01 ^b^
**Controls**				
Ciprofloxacin	69.25 ± 0.03 ^d^	62.45 ± 0.04 ^d^	56.42 ± 0.03 ^f^	51.77 ± 0.03 ^c^
Quercetin	42.57 ± 0.03 ^b,c^	40.66 ± 0.01 ^b,c^	35.15 ± 0.01 ^c,d^	31.81 ± 0.02 ^a,b^
1% DMSO	−3.72 ± 0.04 ^a^	−9.76 ± 0.01 ^a^	−5.06 ± 0.03 ^a^	−8.24 ± 0.02 ^a^

Mean values of triplicate independent experiments ± SD. Comparison of percentage inhibition at MIC value for each treatment against *K. pneumoniae*. Different letters (^a^–^f^) indicate a significant difference at *p* < 0.05 between the different treatments (per column) at the same MIC value.

**Table 4:** Disruption of mature *K. pneumoniae* biofilms formed by various compounds under dynamic and static conditions.

Compounds	Percentage (%) Inhibition of Mature Biofilm Formed under Dynamic Condition (with Shaking)		Percentage (%) Inhibition of Mature Biofilm Formed under Static Condition (without Shaking)	
	*K. pneumoniae* (ATCC BAA-1705)	*K. pneumoniae* (ATCC 700603)	*K. pneumoniae* (ATCC BAA-1705)	*K. pneumoniae* (ATCC 700603)
Alpha-terpinene	18.55 ± 0.02 ^b^	17.22 ± 0.13 ^b^	15.18 ± 0.05 ^b^	12.15 ± 0.03 ^c,d^
Camphene	5.24 ± 0.01 ^a,b^	2.06 ± 0.05 ^b^	4.56 ± 0.01 ^a,b^	4.08 ± 0.05 ^b, d^
Fisetin	14.83 ± 0.02 ^a,b^	12.33 ± 0.02 ^b^	−8.52 ± 0.01 ^a,b^	−32.43 ± 0.02 ^a,b^
Glycitein	8.89 ± 0.01 ^a,b^	−5.71 ± 0.01 ^b^	6.89 ± 0.01 ^a,b^	−12.53 ± 0.01 ^b,c^
Phytol	24.94 ± 0.04 ^b^	25.88 ± 0.00 ^b^	20.32 ± 0.02 ^b^	18.07 ± 0.01 ^d^
**Controls**				
Ciprofloxacin	44.73 ± 0.04 ^c^	51.88 ± 0.00 ^c^	42.24 ± 0.02 ^b^	39.15 ± 0.01 ^e^
Quercetin	−27.08 ± 0.01 ^a^	−44.55 ± 0.01 ^a^	−35.46 ± 0.02 ^a^	−52.25 ± 0.02 ^a^
1% DMSO	−39.01 ± 0.01 ^a^	−58.35 ± 0.01 ^a^	−45.67 ± 0.02 ^a^	−68.25 ± 0.02 ^a^

Mean values are of triplicate independent experiments ± SD. Comparison of percentage inhibition at MIC value for each treatment against *K. pneumoniae*. Different letters (^a^–^e^) indicate a significant difference at *p* < 0.05 between the different treatments (per column) at the same MIC value.

## Data Availability

Not applicable.

## Supplementary Materials

The following supporting information can be downloaded at https://www.mdpi.com/article/10.3390/plants11111429/s1, Table S1: Exopolysaccharide reduction in *K. pneumoniae* (ATCC 700603 and ATCC BAA-1705) by studied phytochemical compounds
