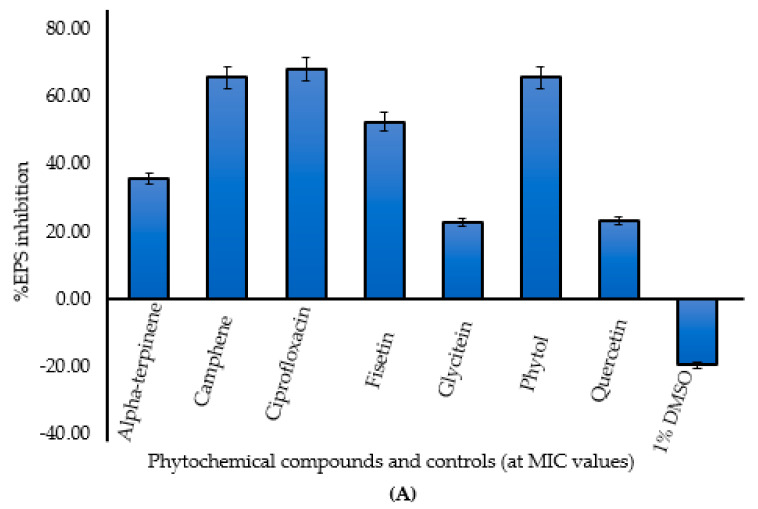# Correction: Adeosun et al. Anti-Biofilm and Associated Anti-Virulence Activities of Selected Phytochemical Compounds against *Klebsiella pneumoniae*. *Plants* 2022, *11*, 1429

**DOI:** 10.3390/plants12061236

**Published:** 2023-03-08

**Authors:** Idowu J. Adeosun, Itumeleng T. Baloyi, Sekelwa Cosa

**Affiliations:** Department of Biochemistry, Genetics and Microbiology, Division of Microbiology, University of Pretoria, Private Bag X20, Hatfield, Pretoria 0028, South Africa

In the original publication [1], there was a mistake in Figure 1A as published. Figure 1A and 1B were the same (Figure 1B was duplicated as both 1A and 1B). The corrected Figure 1A appears below. The authors state that the scientific conclusions are unaffected. This correction was approved by the Academic Editor. The original publication has also been updated.