# Supplementary material for: Anti-Biofilm and Associated Anti-Virulence Activities of Selected Phytochemical Compounds against Klebsiella pneumoniae
Source: Plants (Basel). 2022 May 27;11(11):1429. doi: 10.3390/plants11111429 (PMC9182603; doi:10.3390/plants11111429)
Supplement: Supplementary file 1 [file plants-11-01429-s001.zip › plants-1732664-supplementary.pdf]

Supplementary data

Table S1. Exopolysaccharide reduction in *K. pneumoniae* (ATCC 700603 and ATCC BAA-1705) by studied phytochemical compounds

Compounds	*K. pneumoniae* ATCC 700603		*K. pneumoniae* ATCC BAA-1705	
	EPS Quantity (OD Values)	% of EPS Quantity	EPS Quantity (OD Values)	% of EPS Quantity
Phytol	1.19	34.09	2.41	61.01
Glycitein	2.69	77.08	2.39	60.51
Camphene	1.19	34.09	2.22	56.20
Fisetin	1.65	47.28	2.83	71.65
Alpha-terpinene	2.24	64.18	2.63	66.58
**Controls**				
Quercetin	2.68	76.79	3.42	86.58
Ciprofloxacin	1.05	31.55	2.01	53.74
1% DMSO	4.17	119.48	4.13	104.56
